# Use of a Noninvasive Continuous Monitoring Device in the Management of Atrial Fibrillation: A Pilot Study

**DOI:** 10.1111/pace.12053

**Published:** 2012-12-13

**Authors:** Michael A Rosenberg, Michelle Samuel, Amit Thosani, Peter J Zimetbaum

**Affiliations:** * Division of Electrophysiology, Beth Israel Deaconess Medical Center, Harvard Medical SchoolBoston, Massachusetts; † Division of Electrophysiology, West Penn Allegheny Health System, Temple UniversityPittsburgh, Pennsylvania

**Keywords:** *atrial fibrillation*, *arrhythmias*, *cardiac rhythm monitoring*

## Abstract

**Background:**

*Outpatient ambulatory cardiac rhythm monitoring is a routine part of the management of patients with paroxysmal atrial fibrillation (AF). Current systems are limited by patient convenience and practicality*.

**Methods:**

*We compared the Zio*® *Patch, a single-use, noninvasive waterproof long-term continuous monitoring patch, with a 24-hour Holter monitor in 74 consecutive patients with paroxysmal AF referred for Holter monitoring for detection of arrhythmias*.

**Results:**

*The Zio*® *Patch was well tolerated, with a mean monitoring period of 10.8 ± 2.8 days (range 4–14 days). Over a 24-hour period, there was excellent agreement between the Zio*® *Patch and Holter for identifying AF events and estimating AF burden. Although there was no difference in AF burden estimated by the Zio*® *Patch and the Holter monitor, AF events were identified in 18 additional individuals, and the documented pattern of AF (persistent or paroxysmal) changed in 21 patients after Zio*® *Patch monitoring. Other clinically relevant cardiac events recorded on the Zio*® *Patch after the first 24 hours of monitoring, including symptomatic ventricular pauses, prompted referrals for pacemaker placement or changes in medications. As a result of the findings from the Zio*® *Patch, 28.4% of patients had a change in their clinical management*.

**Conclusions:**

*The Zio*® *Patch was well tolerated, and allowed significantly longer continuous monitoring than a Holter, resulting in an improvement in clinical accuracy, the detection of potentially malignant arrhythmias, and a meaningful change in clinical management. Further studies are necessary to examine the long-term impact of the use of the Zio*® *Patch in AF management*.

## Introduction

Atrial fibrillation (AF) is the most common sustained cardiac arrhythmia, and its prevalence in the population is increasing.^1^ Treatment of AF, regardless of the etiology, is a heavy burden on the healthcare system, with annual U.S. costs for AF management $6.65 billion total, with $2.93 billion for hospitalizations alone.^2^ Due to the frequency of asymptomatic episodes,^3^,^4^ outpatient ambulatory cardiac rhythm monitoring is an integral part of the management of paroxysmal AF. In many cases, clinical decisions such as antiarrhythmic medication dosage adjustment, the need for cardioversion, and potentially the need for anticoagulation, are based on the ability of clinicians to detect and document the presence of recurrence and burden of AF. In addition, outpatient monitoring that is continuous can also reveal the presence of other significant arrhythmias, which necessitate management.

Currently available methods for noninvasive remote cardiac monitoring include the continuous 24-hour Holter monitor, moderate-term (2–4 weeks) patient-triggered event recorders, and mobile cardiac outpatient telemetry. Although each of these monitors has been demonstrated to have utility in the management of AF, as well as in the diagnosis of palpitations,^5^ they are limited by the patient convenience, as many are bulky in size and all require leads. Further, given that monitoring over more than a day or two requires electrode changes and given that current monitors cannot get wet, current monitors are also limited in not practically being able to truly be used continuously for an extended period of time. In this pilot study, we examine a novel outpatient ambulatory cardiac rhythm monitoring device called the Zio® Patch (iRhythm Technologies, Inc., San Francisco, CA, USA), a single-use, noninvasive waterproof long-term continuous monitoring patch, capable of continuously monitoring for up to 14 days, and compare its use with that of the 24-hour Holter monitor in the management of patients with AF. As a new type of continuous monitoring technology, we sought to determine if the Zio® Patch would be well tolerated, function as well as a Holter monitor in the first 24 hours of use in terms of the detection of AF and other arrhythmias, and whether the additional days of monitoring would be tolerated and yield meaningful clinical findings.

## Methods

### Patients

Between April 27, 2011 and May 25, 2012, 75 consecutive patients currently undergoing management of AF at the Beth Israel Deaconess Medical Center, Boston, MA, received both a Zio® Patch as well as 24-hour Holter monitor simultaneously to determine the pattern of AF, to document a response to therapy and to potentially diagnose other arrhythmias. One patient was excluded due to the Zio® Patch inadvertently not being activated during placement, leaving an analysis group of 74 patients. This study was in compliance with the institutional review board of the Beth Israel Deaconess Medical Center, and informed consent was obtained from all patients enrolled.

### Devices and Monitoring Protocols

The Zio® Patch (iRhythm Technologies, Inc.) is a recently-introduced, single-use, noninvasive waterproof continuously recording ambulatory cardiac rhythm monitoring patch that is Food and Drug Administration (FDA) cleared for continuous use for up to 14 days. The Zio® Patch also has an integrated trigger button that can be pressed to make a mark in the continuously recorded data stream to subsequently allow for accurate correlation with symptoms. Patients were instructed to wear the device and press the integrated trigger button when they felt symptoms. The rhythm data were collected on the device, and after the device was received by the manufacturer, the data were analyzed using the manufacturer's FDA-cleared proprietary algorithm and underwent technical review for report creation and quality assurance, after which the report was then uploaded to a secure website, where it was reviewed by cardiologists on the investigation team. All patients were given a 24-hour Holter monitor to wear simultaneously at the time of Zio® Patch attachment, with the same instructions to record any symptoms during this period as well. Holter rhythm data were analyzed by independent clinical cardiologists and technical staff of the Arrhythmia Monitoring Laboratory of the Beth Israel Deaconess Medical Center. Investigators reading the Zio® Patch were blinded to the reports of the 24-hour Holter monitor. Patients were instructed to wear the Zio® Patch for as long as possible, with the goal of obtaining up to 14 days of recording. After completion of the Zio® Patch monitoring period, patients were instructed to mail the Zio® Patch back to the manufacturer for data extraction and analysis. Significant arrhythmias were defined as AF or atrial flutter (these arrhythmias were grouped together to avoid any confusion or issues with ambiguity), other supraventricular tachycardias (not including AF or atrial flutter) for >4 beats, sustained ventricular tachycardia (>4 beats), junctional rhythm, sinus bradycardia (<50 beats/min), and complete or high-grade heart block.

### Analysis

Stata IC 11.0 (StataCorp LP, College Station, TX, USA) was used for all analyses. Continuous variables were tested for normality using a Skew-Kurtosis test prior to analyses, and analyzed using a Student's paired *t*-test for comparisons between the Zio® Patch and Holter. For survival analysis, follow-up time was measured in intervals of days, with time to first arrhythmic event counted from enrollment (day 0) until the day of the episode. Cox regression was used for longitudinal analysis, with follow-up time as time from enrollment until the day of the first episode of AF recorded on the Zio® Patch. Pearson's correlation coefficient was used to examine AF burden, of which the Zio® Patches reported AF burden for the entire time period, as well as in 2-day increments (as such, the comparison with 24-hour Holter burden was performed using a correlation coefficient rather than an intraclass correlation coefficient). A kappa statistic was calculated for the clinical classification of AF into none, paroxysmal (<100% burden), and persistent (100% burden) based on Holter and Zio® Patch monitoring. All authors had access to the data and manuscript as written.

## Results

Table I lists the baseline characteristics for the study population. All patients were referred for Holter monitoring for the evaluation of paroxysmal AF. Of the 74 patients enrolled, 67 had an electrocardiogram performed in the clinic prior to enrollment that was interpreted as sinus rhythm (56 patients) or AF, atrial flutter, or atrial tachycardia (11 patients).

**Table I:** Baseline Characteristics

General	
Age (Mean ± SD)	64.5 ± 8.1
Male sex (N, %)	41 (54.7%)
Caucasian race (N, %)	70 (93.3%)
Medical History	
HTN (N, %)	36 (48.0%)
Diabetes (N, %)	6 (8.0%)
CHF (N, %)	4 (5.3%)
CAD (N, %)	3 (4.0%)
Medications	
*β*-blocker (N, %)	38 (50.7%)
Calcium channel blocker (N, %)	16 (21.3%)
Digoxin (N, %)	0 (0%)
AAD (N, %)	24 (32.0%)
Clinic	
SBP (Mean ± SD)	123.4 ± 18.4
DBP (Mean ± SD)	73.2 ± 8.3
HR (Mean ± SD)	65.2 ± 15.2
Sinus rhythm (N, %)	56 (83.6%)
AF Characteristics	
Symptomatic AF (N, %)	49 (67.1%)
Prior CV (N, %)	16 (21.3%)
Prior PVI (N, %)	10 (10.3%)

AAD = current use of antiarrhythmic medication; AF = atrial fibrillation; CAD = history of coronary artery disease; CHF = history of congestive heart failure; CV = cardioversion (electrical or chemical with ibutilide); DBP = diastolic blood pressure (in mm Hg); HR = heart rate (in beats/min); HTN = history of hypertension; PVI = pulmonary vein isolation; SBP = systolic blood pressure (in mm Hg); SD = standard deviation.

During the first 24 hours, patients wore both the Zio® Patch and a Holter monitor simultaneously. During this period, all 25 AF episodes recorded on the 24-hour Holter (mean monitoring time 22.5 ± 1.8 hours) were identified on the Zio® Patch device, and the estimated AF burden of these episodes recorded in the first 24 hours on both devices were comparable—mean AF burden on Holter was 58.4 ± 42.7% and on the Zio® Patch device it was 54.7 ± 41.2% (r = 0.96, P < 0.0001). (Note that the Zio® Patch estimate was based on AF burden over days 1 and 2.)

After the first 24 hours, patients were instructed to continue to wear the Zio® Patch for as long as possible, up to 2 weeks. The mean time for wearing the Zio® Patch was 10.8 days (standard deviation [SD] 2.8 days), with a range of 4–14 days. The reasons for discontinuation were study completion (49 patients), device falling off (16 patients), patient's decision to remove the device (six patients), battery malfunction (one patient), unknown (one patient), and need for other cardiac intervention/testing (one patient). Among patients in whom the device fell off, the mean time worn was 7.9 days (standard deviation 1.8 days, range 5.8–12.2 days). In total, 454 patient-days were recorded using the Zio® Patch, during which time additional AF episodes were diagnosed in 43 patients (58.1%). The incidence rate of AF in this population was 0.095 events/person-day, and the median time to detection of an AF episode was 1 day (range 1–12 days). In patients without AF on clinic ECG or 24-hour Holter monitor, the median time to detection was 3.7 days (SD 3.0 days), with 90% of first AF events detected by day 7. There was no significant difference in detection between patients with and without symptoms with AF (hazard ratio [HR] 1.07 without symptoms, confidence interval [CI] 0.56–2.1, P = 0.84), in older patients (HR 0.99 per year, CI 0.96–1.03, P = 0.74) or in any particular gender (HR 1.14 for men, CI 0.62–2.10, P = 0.66).

As a result of a longer monitoring time, AF episodes were detected in significantly more patients (18) on the Zio® Patch compared with the Holter monitor (P < 0.0001). The estimated AF burden (percent of time in AF) was available in all 43 patients with AF on the Zio® Patch, and was 28.4% (SD 31.2%) in total. The AF burden was estimated in 21 patients (of 25 with AF) wearing the 24-hour Holter, and was 58.4% (SD 42.7%). Overall, among the patients with any AF during the study, there was good correlation between the AF burden estimated by the Zio® Patch and Holter (r = 0.82, P < 0.0001). The clinical classification of the AF pattern changed as a result of wearing the Zio® Patch in 21 patients (Table II), although in general there was agreement between the two devices (kappa 0.49 ± 0.08, P < 0.01).

**Table II:** Change in Clinical Classification of AF Based on Zio Patch Findings

		Zio® Patch				
			
		None	Paroxysmal	Persistent	Total
	None	32	17	0	49
Holter	Paroxysmal	0	12	0	12
	Persistent	0	4	5	9
	Total	32	33	5	70

Four patients without estimates for AF burden on Holter are excluded. See text for details.

The Zio® Patch, like the Holter, has a trigger button to correlate symptoms such as palpitations or dizziness with rhythms, and patients were instructed to press the Zio® Patch trigger button when they felt such symptoms. Overall, 41 patients (55.4%) triggered the device for symptoms, with a mean number of 7.4 symptomatic events per patient reported (SD 10.4, median 3, range 1–60; Tables III and IV). Of the 305 total triggered events, most (200) correlated with sinus rhythm (66%), 87 (29%) correlated with AF, 14 (5%) with supraventricular tachycardia, and four (1%) with pauses. Ten patients reported symptoms that only correlated with AF episodes, while 15 patients reported symptoms that only correlated with sinus rhythm (no arrhythmias recorded). In the 33 patients who were previously designated as having “symptomatic AF,” only 39.5% (SD 43.8%) of triggers were correlated with AF, while 51.5% (SD 42.6%) of triggers correlated with sinus rhythm.

**Table III:** Symptoms/Triggers Reported

Number of Episodes Per Patient	Number of Patients
0	33 (44.6%)
1–5	24 (32.4%)
>5	17 (23.0%)

**Table IV:** Heart Rhythms Reported with Triggered Episodes

Rhythm	Percent of Triggers
Sinus rhythm	55.2% (SD 43.2%)
AF	37.6% (SD 43.7%)
SV ectopy	25.1% (SD 33.8%)
Ventricular ectopy	20.6% (SD 30.5%)
SVT	4.8% (SD 17.2%)
VT	0 (SD 0%)
Pauses (>3 seconds)	2.4% (SD 15.6%)

AF = atrial fibrillation or flutter; SV = supraventricular; SVT = supraventricular tachycardia (>4 beats); VT = ventricular tachycardia (>4 beats).

**Figure 1:**
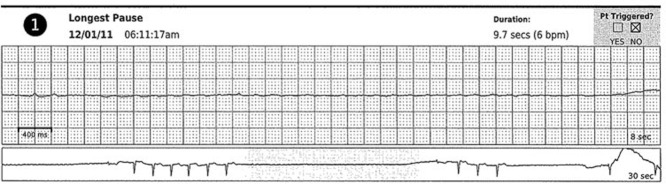
*Zio*® *Patch recording from a patient with a 9.7-second pause. Note that the patient did not report any symptoms with the pause. No pauses were recorded on the 24-hour Holter monitor. This patient was referred for permanent pacemaker placement*.

In addition to AF episodes, the Zio® Patch also identified ventricular (18 patients, 24.3%, range 1–92 per patient) and supraventricular (25 patients, 33.8%, range 1–336 per patient) tachycardias of >4 beats, pauses (four patients, 5.4%, range 1–99 episodes/patient, duration 3.1–9.7 seconds), and episodes of atrioventricular block (one patient, 1.4%, one event, Mobitz 1 Wenckbach). Two patients had pauses of over 5 seconds and were referred for pacemaker placement. Of these, one had pauses also recorded on the 24-hour Holter, while the other, with pauses of up to 9.7 seconds, had no pause events recorded on the 24-hour Holter (Fig. 1).

As a result of completing the Zio® Patch study, 21 patients (28.4%) had a change in their management. The most common change was a change in antiarrhythmic medication in 13 patients. A change in anticoagulation status (either starting or stopping oral anticoagulation) occurred in four patients. Other changes included: recommendation of placement of a pacemaker in two patients, recommendation for atrioventricular junction ablation in one patient, recommendation for pulmonary vein isolation procedure in one patient, and recommendation for cardioversion in two patients.

## Discussion

The Zio® Patch device is a novel, single-use, noninvasive waterproof continuously recording ambulatory cardiac rhythm monitoring patch that is well tolerated, and in this single-center pilot study, was shown to be superior to a 24-Holter monitor for detection of AF episodes and other significant cardiac arrhythmias. Although larger studies are needed to make broad generalizations about the nature of AF detection and characteristics of AF in specific populations, several observations from this pilot study can be made about the use of continuous monitoring of AF using the Zio® Patch.

For one, we found that most recurrence of AF in this population was detected within the first week of wearing the Zio® Patch, although a small number were also detected after up to 12 days of monitoring. This finding implies that for patients in whom recurrence detection is the goal of monitoring, a week of monitoring may be sufficient, although further study is needed.

Second, we observed that while in general there was good agreement between the 24-hour Holter monitor and the Zio® Patch in terms of capturing the recurrence pattern of AF (paroxysmal or persistent), 21 patients were reclassified after longer term continuous monitoring with the Zio® Patch. This more specific characteristic of the AF pattern may have important implications for rate or rhythm management.

Third, we confirmed the recognized finding that many episodes of perceived AF are in fact sinus rhythm. It is well described that many episodes of AF, even in patients who have previously reported symptoms with recurrence, occur without symptoms,^6^,^7^ and these results further support the need for electrocardiographic monitoring in addition to clinical follow-up in the management of AF. The ability to identify asymptomatic AF is particularly important in patients who have undergone AF ablation, where AF recurrence is often asymptomatic.^8^,^9^

Finally, we observed that several clinically significant arrhythmias, such as the 9.7-second pause detected in one patient, were found only with continuous monitoring for longer periods, and that reliance on symptoms alone was inadequate for diagnosing these rhythms. Other studies have also found other malignant rhythms with monitoring for AF.^5^ Importantly, we detected no clinical impact from the lack of real-time detection inherent in the Zio® Patch monitoring in the diagnosis of these arrhythmias, and patients were referred for appropriate management. However, larger studies are necessary to fully establish the safety of the Zio Patch in arrhythmia detection.

The need for convenient, well-tolerated, cost-effective cardiac monitoring for AF is likely to increase as AF becomes more prevalent.^1^ Long-term outpatient ambulatory cardiac rhythm monitoring provides objective data regarding AF recurrence and burden, as well as the presence of other clinically significant arrhythmias, and is necessary for decisions about long-term management. Further, recent clinical studies have demonstrated that even shorter durations of AF than 48 hours^10^,^11^ may be clinically significant.^4^,^12^,^13^ Recently, Healey et al. showed that episodes of AF of as brief duration as 6 minutes were associated with an increased risk of stroke as well as future clinical AF.^4^ These studies suggest that future clinical decision making could be influenced by the ability to objectively and accurately detect AF episodes, and thus the Zio® Patch adds to the options for long-term outpatient ambulatory cardiac rhythm monitoring.

In summary, in this single-center pilot study, we found that the Zio® Patch was superior to a 24-hour Holter with regard to ability to detect AF episodes, and was comparable in the ability to quantify AF burden, with the additional benefit of detecting other more malignant arrhythmias due to the longer duration of detection leading to clinically meaningful changes in clinical management. The Zio® Patch was well tolerated by most participants, with a median follow-up time of over a week. Although larger studies will be necessary to determine the efficacy of the Zio® Patch device in terms of overall arrhythmia detection and cost-effectiveness compared with other outpatient ambulatory cardiac rhythm monitoring devices, the promising data from this pilot study suggests that the Zio® Patch device may represent a more convenient and efficient method of outpatient arrhythmia detection than current methods using Holter monitors, event recorders, or mobile cardiac outpatient telemetry.
